# Application of Sustainable Procurement Policy to Improve the Circularity of Construction and Demolition Waste Resources in Australia

**DOI:** 10.1007/s42824-022-00069-z

**Published:** 2022-10-29

**Authors:** Salman Shooshtarian, Tayyab Maqsood, Peter S. P. Wong, Louis Bettini

**Affiliations:** 1 School of Property, Construction and Project Management, RMIT University, Melbourne, Australia; 2 Western Australian Main Roads, Perth, Australia

**Keywords:** Construction and demolition waste, Circular economy, The built environment sector, Resource efficiency, Australia

## Abstract

The sustainable management of construction and demolition (C&D) necessitates efficient waste minimisation policies. Sustainable procurement of recycled waste products (RWPs) is an encouragement-based policy approach that can serve this purpose. This policy aids stakeholders in enhancing the circular economy (CE) in the built environment (BE) sector. However, this procurement method is yet to be successfully adapted globally or in Australia. Hence, this study was conducted to shed light on the use of sustainable procurement in the Australian BE sector. Based on a mixed-methods methodology, 49 relevant academic, industry and government publications were analysed during the review process. The findings reveal that the two most significant obstacles are the lack of supportive organisational culture and uncertainty about RWP quality. In addition, implementing clear and supporting regulations and maintaining transparency and good governance are identified as the two most important enablers. A model was proposed to facilitate the adoption of sustainable procurement, based on the research findings. The study includes a number of suggestions to encourage the acquisition of C&D RWPs for construction projects. This review is anticipated to contribute to three areas: sustainable procurement theory, policy development, and BE sector practice.

## Introduction

In the built environment (BE) industry, environmental and ecological problems linked with building activities have become a growing concern. The substantial amount of trash generated on construction sites is cited as a significant obstacle to the global expansion of the BE industry (Aslam et al. 2020; Bao and Lu 2020; Shooshtarian et al. 2021b). Relevant literature has highlighted the importance of a policy approach to the sustainable management of this waste stream (Alhola et al. 2019; Shooshtarian et al. 2021d). Sustainable procurement, among existing rules, has the potential to improve construction and demolition (C&D) waste management systems. This policy strategy will also promote resource circularity in the BE industry. Multiple organisations define sustainable procurement in various contexts and applications. For instance, the United Nations (UN), the British government and the Australasian Procurement and Construction Council (APCC) recognise the following definition: ‘*A process whereby organisations meet their needs for goods, services, works and utilities in a way that achieves value for money on a whole life basis in terms of generating benefits not only to the organisation, but also to society and the economy, whilst minimising damage to the environment’* (cited in Commonwealth of Australia 2013, p.4). Other terms that define the concept of sustainable procurement include ‘green public procurement’, ‘sustainable public procurement’, ‘environmental purchasing’, ‘circular procurement’ and ‘environmentally preferable procurement’. Table 1 displays the variations in definitions of sustainable procurement.

**Table 1:** Variations of sustainable procurement and their definitions

Concept	Definition	Source
Sustainable sourcing	Managing all aspects of the upstream elements of the supply chain to maximise the triple bottom line which entails social, economic and environmental performance	Pagell and Shevchenko (2014)
Green public procurement	Procuring goods, services and works with a reduced environmental impact throughout their life cycle when compared to goods, services and works with the same primary function that would otherwise be produced	Diófási and Valkó (2014)
Circular procurement	A process that enables the purchasing party to ensure that, at the end of their service life or useful life, products or materials will be re-used effectively in a new cycle, where the products and materials crucially retain their quality and value It is the process in which a product, a service or a project is purchased according to the principles of a circular economy. In this process, the technical aspects of the product are as circular as possible, taking maintenance and return policies at the end of the use period into account, as well as including financial incentives to guarantee circular use	PIANOo (2020) Van Oppen et al. (2018)
Environmental purchasing	The inclusion of environmental factors in decisions on the purchase of products and/or services	Australian Government (2003)
Environmentally preferable procurement	It involves purchasing products or services that have a lesser or reduced effect on human health and the environment when compared with competing products or services that serve the same purpose	NCDENR (2010)

For many years, the economic component was the most important indicator in the process of public procurement. In response to the strain on natural resources and heightened public awareness of the negative consequences of human activities on the environment, governments and corporations have shifted their procurement practices towards greater environmental sustainability. Sustainable procurement incorporates specifications, requirements and criteria that are compatible with environmental and social protection. This policy approach ‘*is consistent with the principles of sustainable development, such as ensuring a strong, healthy and just society, living within environmental limits, and promoting good governance*’ (Brammer and Walker 2011, p.128). Furthermore, sustainable procurement supports the application of circular economy (CE) principles and cradle-to-grave design in BE sector. Several benefits of sustainable procurement application are identified in previous literature. For instance, Pick (2017) reported benefits such as stimulating the local economy, contributing to the brand of ‘zero waste’ and other sustainability goals and reducing municipal operating costs by selecting durable and reusable materials.

In prior research, several benefits of sustainable procurement application have been established. For instance, Pick (2017) reported that sustainable procurement stimulates the local economy, contributing to the concept of ‘zero waste’ and other sustainability goals and reducing municipal operating costs by selecting durable, recovered and reusable materials.

Implementing sustainable procurement practices in the BE sector has various environmental benefits. It could, for instance, result in a reduction of greenhouse gas (GHG) emissions by decreasing energy use. This is significant since, according to estimates, around 80% of the energy required to manufacture a building is utilised to produce and transport building materials. In addition, sustainable procurement contributes to an increase in waste recovery and creates a demand for C&D recycled waste products (RWPs) with a small environmental footprint. A study indicated that compelling suppliers to agree to waste reduction goals is one of the five essential roles of sustainable procurement policies in 25 studied countries (Brammer and Walker 2011). According to a Dutch study (Zhang et al. 2020), sustainable procurement is the major strategy for promoting concrete recycling. Additionally, it was reported that sustainable procurement is the most viable strategy for building a long-term system for utilising RWPs in the construction industry in China (He et al. 2014).

The substantial amount of waste generated by the BE sector implies that this strategy will have a considerable impact on the environment. Sustainable procurement has the potential to influence local producers and encourage the development of sustainable products and practices by acting as a demand-side market force. Consequently, it provides substantial environmental and social benefits related to resource efficiency, managing operational costs, increasing compliance with environmental legislation, addressing environmental concerns and generating end markets for C&D waste products (Ershadi et al. 2021). This is significant in light of two key events that have recently impacted the waste recovery industry: COVID- 19 (Caldera et al. 2022; Shooshtarian et al. 2022a) and new regulations in some countries (particularly China) to prohibit the import of waste from industrialised nations (Shooshtarian et al. 2021d). In the last two decades, efforts to operationalize sustainable procurement methods have gained steam. In 2007, the UN Secretary-General vowed to create a sustainable United Nations. Since then, a number of initiatives have built a new procurement policy framework. In response, the UN Environment Program (UNEP) established a sustainable UN section to manage the sustainability performance of UN organisations. Four years later, UNEP and the International Labour Organization (ILO) issued ‘buying for a better world’, a manual on sustainable procurement for managers and practitioners (Sustainable United Nations 2011). The fundamentals of International Organization for Standardization (ISO) 20,400 (International Organization for Standardization 2017) are intended to assist an organisation in understanding the context and the drivers and establishing a sustainable procurement strategy that is aligned with the context and the drivers. Shaun McCarthy, from Action Sustainability, one of the organisations that contributed to the creation of ISO 20400, commented: *“societal expectations are at an all-time high. It is no longer acceptable to do a few sustainability things in your organisation and ignore your supply chain. This standard can be a game-changer if implemented the way it was designed to be used*” (cited in Peretti and Druhmann 2021, p. 35). Table 2 outlines universally applicable sustainable procurement guidelines.

**Table 2:** International guidelines for sustainable procurement

Organisation	Guideline	Focus
ISO	ISO 204001 (2017)/sustainable Procurement—Guidance	It provides guidelines for integrating sustainability into an organisation’s procurement processes. It covers various stages of the procurement process, outlining the steps required to integrate social responsibility into the purchasing function
ISO	ISO 14001 (2015)/ Environmental Management	Its major focus is on the structure, implementation and maintenance of a formal environmental management system
UNEP	Buying for a Better World (Sustainable United Nations 2011)	It aims to (1) concrete and valid arguments for the UN to engage in sustainable procurement, (2) provide recommendations on the development of a sustainable procurement action plan and (3) develop guidance on the integration of sustainable development principles in the UN procurement cycle
Building Research Establishment’s Environmental Assessment Method (BREEAM)	BRE Group (2018)	Refers extensively to sustainable procurement and incorporates ISO 20400 as the recommended sustainable procurement strategy (through providing additional credits where ISO 20400 has been adopted)

### Literature Review: Global Context

Analysis of sustainable procurement policies, practices and experiences in a global context provides motivations for those states and organisations seeking to establish CE and achieve sustainable outcomes. At the national and international levels, figures indicate an increase in applying this policy approach under various names. For instance, in South Korea, a study shows that all public institutions’ total expenditure on green products increased from USD 759 million in 2006 to USD 2945 million in 2017 (Gill 2019). In addition, reports indicate that the extent to which this policy’s associated benefits are being realised has expanded globally. For instance, in the European Union (EU), sustainable procurement represents 16% of the member countries’ gross domestic product (GDP). Whereas, in the case of Organisation for Economic Cooperation and Development (OECD) countries, it varies between 8 and 25% (Da Costa and Da Motta 2019). In the US, the Environmentally Preferable Purchasing (EPP) program has generated high cost and environmental benefits, including the purchase of nearly 7 million Electronic Product Environmental Assessment Tool (EPEAT)-registered products by the federal government in 2018, resulting in cost savings of around $182.5 million (US EPA 2021). As a global leader in public procurement practices, China launched Green Public Procurement (GPP) in 2004. However, the literature reported that unclear regulatory requirements, costs associated with GPP implementation and procurement professionals’ low awareness of GPP benefits hinder GPP expansion in China (Geng and Doberstein 2008; Wang et al. 2011).

In the context of the BE sector, the literature provides scant information on sustainable procurement performance, particularly in the area of waste diversion from landfills. Multiple research publications describe the implementation of sustainable procurement without quantitative evidence of its efficacy (Aboginije et al. 2020; Tan et al. 2011). For instance, in France, by including green public procurement into its Energy Transition for Green Growth Act 2015 (Nicklaus and Kochert 2015), the French government aimed to boost the use of recycled aggregate (RA) in road construction projects by 60% by 2020. In Finland, the BE’s sustainable procurement target is set at 29% (KEINO 2019). Some sustainable procurement policies in South Korea have encouraged the acquisition of certified RWPs, particularly in the BE sector (Gill 2019). In Canada (Vancouver), research from the business sector indicates that a significant number of Canadian municipalities have begun implementing sustainable procurement policies for civil construction projects (Reeve Consulting 2012). However, the following research revealed that the implementation of sustainable procurement has mostly fallen short of its anticipated objectives due to a lack of funding (Pick 2017; Ruparathna and Hewage 2015). In Malaysia, the BE sector’s implementation of sustainable procurement is yet in its infancy and acceptance is dispersed and fragmented (Bohari et al. 2017). In China, sustainable procurement mandating the use of at least 15% RWPs in public projects is anticipated to result in the reuse of more than 1.1 million m^3^ of reclaimed bricks and 1 million tonnes (Mt) of crushed recycled concrete (CRC) (He et al. 2014).

### Research Rationale and the Gap in the Australian Context

Australia has witnessed a significant rise in C&D trash output and recycling in the building and engineering sector (National Waste Report 2020). Sustainable procurement must be the focal point of waste management planning in the Australian built environment in order to ensure minimal waste landfilling. For this reason, it must be properly comprehended, which includes identifying potential hurdles and enablers, studying its realised impacts and relevant activities and analysing its local and national regulations. This approach is recommended in numerous Australian waste-related recommendations and strategy documents, including the National Waste Report (Shooshtarian et al. 2020b). In Australia, there is a dearth of scientific study on sustainable procurement in the built environment.

In recent years, this problem has been the subject of a small number of research; nonetheless, their breadth was limited. The limitations pertain to the study’s focus or the chosen research methodology. Specifically, carbon emission reductions (Lingegård et al. 2021), COVID-19 (Caldera et al. 2022), a focus on certain stakeholders (Ershadi et al. 2021) and market development (Caldera et al. 2020; Shooshtarian et al. 2020a) have been studied in the past. Using research methods such as case study inherited constrained generalisability (Sanchez et al. 2014) offers limited information regarding sustainable procurement in the BE sector. An earlier literature review (Shooshtarian et al. 2022c) indicates that the relationship between procurement and C&D waste minimisation in Australia has not been thoroughly investigated. Multiple studies demonstrate the need to evaluate the effect of procurement strategies on waste management planning (Park and Tucker 2017; Udawatta et al. 2015b). Moreover, the exposure of previous writers to real-time concerns stated by stakeholders in their research projects has revealed such deficiencies in the Australian BE sector’s CE and waste management policy and practice. Survey analysis (Ershadi et al. 2021; Shooshtarian et al. 2021a), a series of interviews (Ershadi et al. 2021; Shooshtarian et al. 2021a), specialised workshops and industry seminars (SBEnrc P.175 2021) and other personal communications captured the concerns of stakeholders. In light of the preceding, this research was done to fill this gap and provide a deeper understanding of this policy approach.

The review findings are expected to contribute to the theory and practice of sustainable procurement policy implications in the BE sector. This research is timely as the Australian federal government is seeking to maximise the benefits of sustainable procurement nationally. Lastly, the BE sector has embarked on the journey to gradually adopt a CE (Shooshtarian et al. 2021b), which in part requires knowledge of sustainable procurement. This research is part of a larger national research project that seeks to improve waste market development for the C&D waste stream (SBEnrc P.175 2021).

This paper presents the first review of its kind in the Australian context. It is anticipated that the review’s findings will add to the theory and practise of sustainable procurement policy implications in the BE sector. Furthermore, this research is timely, since the federal government of Australia seeks to maximise the national advantages of sustainable procurement. Last but not least, the BE industry has begun to adopt a CE (Shooshtarian et al. 2021b), which demands an understanding of sustainable procurement. This research is a part of a wider national research project that aims to enhance the growth of the C&D waste stream’s waste market (SBEnrc P.175 2021).

### Research Aims and Objectives

This research focuses on increasing the use of RWPs in Australia by employing a sustainable procurement strategy. The aim of the study was to examine the current condition of sustainable procurement in BE sector’s waste management. The following objectives were established to accomplish the research aim:

1. To identify sustainable procurement principles to guide policy development
2. To explore potential barriers and enablers of sustainable procurement in the BE sector
3. To analyse existing national and territorial rules, policies and guidelines that support sustainable procurement
4. To analyse sustainable procurement practical outcome
5. To develop a model to facilitate the successful implementation of sustainable procurement in the BE sector

## Methods

### Study Context

Waste management and resource recovery activities in Australia are generally governed at the state and territory levels. Each jurisdiction has a single public agency responsible for regulating waste-related activity. Due to the huge population and intensive construction activity, the most C&D waste is generated in five Australian major states: New South Wales (NSW), Victoria (Vic), Queensland (Qld), Western Australia (WA) and South Australia (SA) (Australian Bureau of Statistics 2022). In 2018–2019, the BE sector produced around 27 Mt of C&D waste, or 44% of the nation’s total core waste. During the 13-year period for which data is available, C&D waste generation per capita increased by 32%. Of 27 Mt of C&D waste, 6.5 Mt and 20.5 Mt were disposed of and reclaimed, respectively (recycled and energy recovered) (National Waste Report 2020). These numbers indicate a large opportunity for sustainable procurement in this waste stream.

### Research Design

**Fig. 1:**
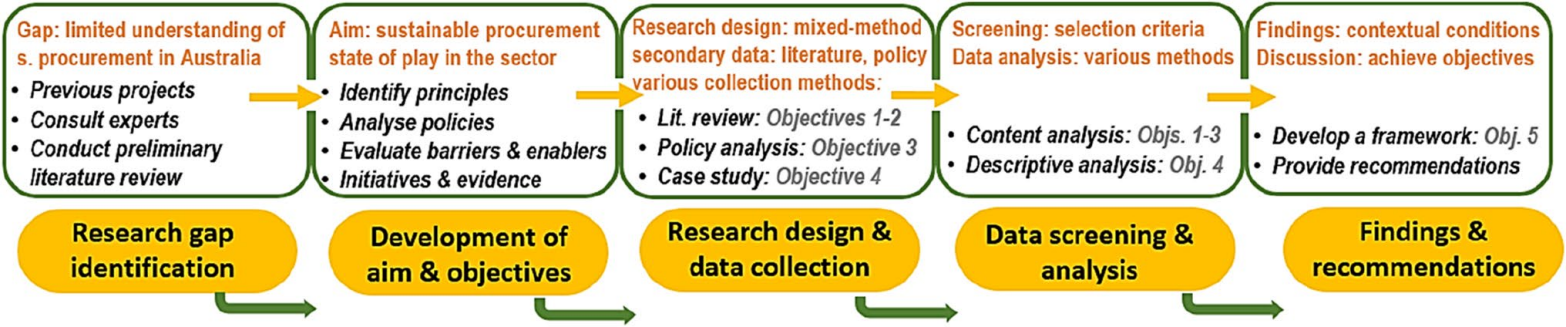
Overview of the research process in this study

A mixed-method approach was adopted to address the five study objectives using qualitative and quantitative secondary data. To apply this research approach, multiple data collection methods suitable for each objective were used. Using multiple data collection methods in construction management research is proven to offer a wide range of benefits (Axinn and Pearce 2006; Fellows and Liu 2021); predominantly, it is realised through the provision of more substantial evidence and more confidence in research findings. The findings from the first four objectives were used to create a model guiding effort to implement sustainable procurement in the BE sector (Objective 5). Figure 1 displays an overview of the research process undertaken in this study.

### Data Collection and Analysis

As illustrated in Fig. 1, a literature review method was employed to understand sustainable procurement principles (Objective 1), challenges and drivers (Objective 2). The primary data sources included academic literature, industry and government reports and other non-conventional resources such as company websites and project catalogues. The keywords used in the desktop research were ‘sustainable procurement’, ‘green procurement’ (and other alternatives), ‘government purchasing power’, ‘built environment’, ‘C&D waste’, ‘CE’ and ‘the construction industry’. In Australia, the use of RWPs through sustainable procurement guidelines was introduced just recently, and its practical application does not have a long history. Therefore, it is expected that there would be little evidence demonstrating its suitability in the Australian context with regard to C&D waste end markets operation and size. This study, however, sought to understand how this waste management strategy impacts overseas C&D waste end-markets. To address this issue, in Objective 2, the analysis of the international studies was conducted to understand the main challenges and enablers of sustainable procurement in the worldwide experience. The selected resources for this analysis met the following criteria:

- Academic literature written in English and published within the last decade (2013–2022)
- Policies and guidelines that are currently in effect
- Recourse with research scope defined only for the BE sector
- Findings were based on empirical evidence including an analysis of Australian construction projects completed in the last two decades (2003–2022).

A case study was conducted to give compelling evidence for the positive outcome within the local context (Objective 4). Two sets of case studies were selected to address this objective. Firstly, four recently established case studies (i.e. Buy Recycled, ASPIRE, ecologiQ and Waste Forum) were selected as successful examples of government-funded initiatives aiming to improve the operation of sustainable procurement and enhance resource circularity in two Australian states. i.e. Vic and WA. Secondly, the utilisation of RWPs across the country was assessed using construction project cases.

For the third objective, policy analysis was utilised to demonstrate how the Australian policy environment supports the implementation of this policy approach in the BE sector. For the policy analysis, the most recent versions of national and state sustainable procurement guidelines, waste strategy documents and pertinent policies were utilised. A case study was conducted to provide compelling for the successful outcome within the local context (Objective 4). To achieve this objective, two sets of case studies were selected. Initially, four recently established case studies (Buy Recycled, ASPIRE, ecologiQ, and Waste Forum) were chosen as successful examples of government-funded initiatives aiming to improve the operation of sustainable procurement and enhance resource circularity in two Australian states: Vic and WA. Secondly, the utilisation of RWPs across the nation was evaluated using examples from construction projects. In terms of data analysis, a variety of analytical models were utilised. The collected literature was subjected to content analysis in order to pick the most representative sustainable principle for the Australian context and to identify the significant challenges and drivers. Content analysis was also applied to national and local government documents. Lastly, descriptive analysis was applied to demonstrate how RWPs were utilised in various Australian construction projects.

## Results

### Descriptive Findings

In total, 49 pieces of literature were identified to meet the selection criteria mentioned above. These include 27 government documents, 11 journal articles, six industry and private organisations’ reports, two conference papers, two research theses and one book chapter. More than 64% of the literature was published within the last five years.

### Sustainable Procurement Significance, Benefits and Principles in the Australian Context

**Fig. 2:**
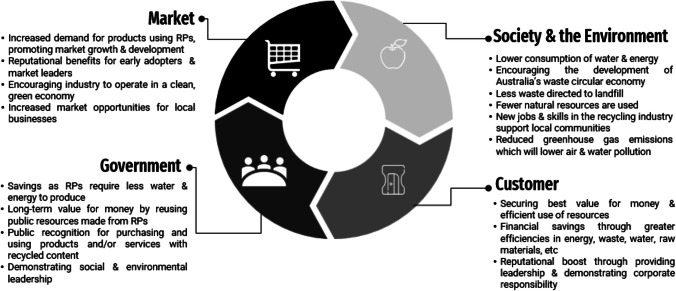
The perceived benefits of sustainable procurement by government, market, society and environment. Source: adapted from (Australian Government, 2020b; Australian ISO 20400 Committee 2018)

The concept of sustainable procurement has piqued the interest of the leading players in the BE industry, according to anecdotal evidence. This section provides a summary of the opinions of Australians regarding the significance, benefits and guiding principles of sustainable procurement for C&D waste management. Involvement of government agencies in the broader application of sustainable procurement practices through the development of specifications, accreditation, quality assurance and raising awareness of the implications of the RWPs (i.e. financial, social, ethical and environmental) and services would be advantageous for boosting the market demand for recovered C&D materials (Hyder Consulting Pty Ltd 2011). According to the results of a survey study (Shooshtarian et al. 2020a), this policy approach, investment in technology and infrastructure and landfill levies are three important factors that have a substantial impact on RWP market development. Another study (Davis et al. 2019) reveals that the BE sector’s stakeholders regard sustainable procurement as a crucial solution for organisational waste management if it is a viable choice or the client agrees to pay more. In a recent investigation, Ershadi et al. (2021) identified eight major areas in which this policy’s benefits can be realised. These include strategic analysis, target formulation, task assignment, planning support, support for tendering, consistency maintenance, operation support and post-review. In 2020, the Australian government issued a new edition of the Sustainable Procurement Guideline, succeeding the 2010 version 2020 (Australian Government 2020b). This document provides a framework for the Australian government to build on efforts to improve sustainability outcomes and mainstream sustainability principles in future procurement. This document provides a list of benefits to the purchaser (government), market, society and the environment that is achieved through buying RWPs. Figure 2 integrates this list with the model proposed by the Australian ISO 20400 Committee.

**Fig. 3:**
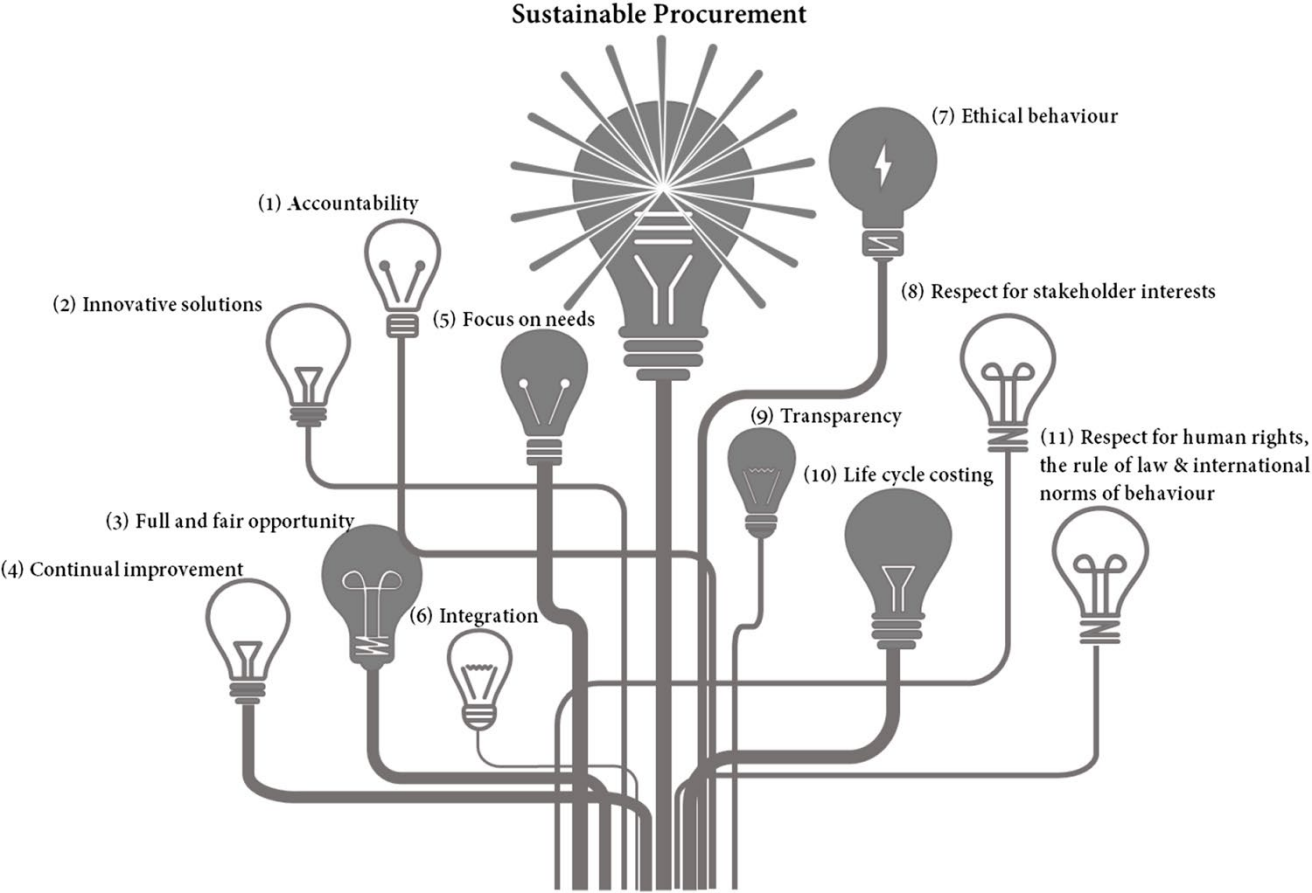
Primary principles of sustainable procurement. Source: adapted from Local Government NSW (2021)

To determine the fundamental principles and functions of sustainable procurement in the Australian context, relevant literature was analysed. The conclusion of the analysis demonstrates that sustainable procurement standards are theoretically supported by eleven principles (Fig. 3). These principles pertain to many facets of sustainable procurement and can serve as a guide for public and private organisations to build their own sustainable procurement strategies. These principles are described in Table 3, and for each principle, a suggested action (in the final column) is provided to transform the principle into sustainable procurement of RWPs in the BE context (Fig. 3).

**Table 3:** The functionality of principles of sustainable procurement and their relevance to RWPs procurement

No	Principle	Description	Action relevant to RWPs procurement
1	Accountability	be accountable for its impacts on society, the economy and the environment, including the impacts of the organisation’s supply chain	To increase the price of using virgin resources in order to hold end-users accountable for their procurement policies and practises
2	Innovative solutions	seek solutions to address its sustainability objectives and encourage innovative procurement practices to promote more sustainable outcomes throughout the entire supply chain	To design policies that promote innovation in the acquisition of RWPs, hence increasing their adoption
3	Full and fair opportunity	avoid bias, and prejudice in all procurement decision making	To prevent PWRs from being compared to virgin materials
4	Continual improvement	work towards continually improving its sustainability practices and outcomes, and encouraging its supply chains to do the same	To optimise the BE sector’s supply chain by integrating RWPs into procurement strategy and processes
5	Focus on needs	review demand, buy only what is needed and seek more sustainable alternatives first	To prioritise RWPs in material specifications for low-value applications
6	Integration	ensure that sustainability is integrated into all existing procurement practices to maximise sustainable outcomes	To encourage the BE sector to increase its capability for sustainable procurement
7	Ethical behaviour	behave ethically and promote ethical behaviour throughout its supply chains	To monitor waste handling and processing activities to reduce the possibility of acquiring RWPs
8	Respect for stakeholder interests	respect, consider and respond to the interests of stakeholders impacted by its procurement activities	To provide a level playing field for all stakeholders involved in the use of RWPs
9	Transparency	be transparent about its procurement processes and how its decisions and activities impact the environment, society and the economy	To maintain transparency in handling, processing and procurement of RWPs
10	Life cycle costing	consider the cost incurred, the value for money achieved and also the costs and benefits on society, the environment and the economy resulting from its procurement activities	To consider the social and environmental advantages of acquiring RWPs
11	Respect for human rights, the rule of law and international norms of behaviour	be aware of any violations throughout its supply chains and actively encourage its suppliers to do the same	To lessen the demand for virgin resources by procuring RWPs

Source: Adapted from Local Government NSW (2021)

### Barriers

Barriers play an important role in moulding sustainable procurement practices and the variation in the outcome of sustainable procurement. The literature has provided various factors that hinder the promotion and application of sustainable procurement initiatives. The nature of issues is heavily context-specific, while there could be similarities among different contexts. Table 4 summarises studies investigating sustainable procurement barriers in developed and developing nations. This table listed 30 barriers identified in the literature. The result of the analysis showed that the two major barriers are the lack of supportive organisational culture and uncertainty about the RWPs quality.

**Table 4:**
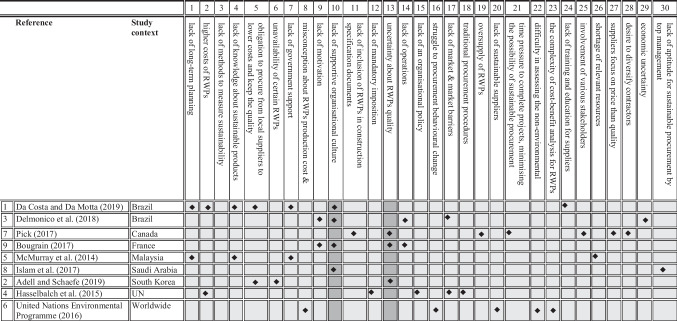
Summary of barriers to applying sustainable procurement in the BE sector

### Enablers

Experts in procurement suggest that sustainable procurement planning is contingent on the fulfilment of a series of mostly context-specific constraints. Therefore, this study explored the main enablers of sustainable procurement. As shown in Table 5, previous research has identified a variety of enabling factors, primarily in the public sector of developed and developing nations. Of the twenty enablers identified in this review, ‘implementing clear and supporting regulations’ and ‘maintaining transparency and good governance’ are the two most important.

**Table 5:**
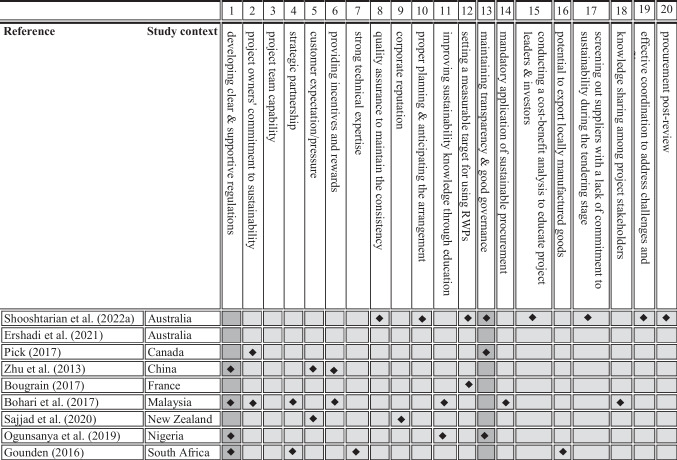
Summary of enablers of applying sustainable procurement in the BE sector

### Review the Existing Sustainable Procurement Policies in Australia

As previously stated, waste regulation in Australia occurs at the state level. However, certain policies of national concern, such as sustainable procurement policies, are developed at the national level. In this section, policy analysis is therefore undertaken at both the national and state levels. In general, strategies for sustainable procurement produced by various governmental bodies are based on a combination of considerations. These include the necessity to future-proof themselves largely in relation to supply constraints, the capacity to meet the demand of emerging markets, cost pressures and the capacity to lower these through energy usage and waste reduction (Ecovadis 2021). This section examines the procurement policies and principles governing sustainable procurement in Australia.

#### National Guidelines and Policies

Regarding waste management, numerous national policies and guidelines have emphasised the environmental impact of government buying practices (Table 6). For instance, the National Strategy for Ecologically Sustainable Development (ESD) (Australian Government 1992), the National Waste Policy (2018) and other sustainable procurement policies significantly support the reuse of RWPs.

**Table 6:** Sustainable procurement in national guidelines and policies

Guideline policy	Issuing organisation	Increased usage of RWPs through sustainable procurement
2020 Sustainable procurement Guide (Australian Government 2020b)	Australian Government - Department of Agriculture, Water, and the Environment	Strategy 8 (sustainable procurement by governments) and Strategy 9 (sustainable procurement by businesses and individuals)
2019 National Waste Policy Action Plan (Australian Government 2019)	Australian Government	Under Target 4 of the National Waste Policy Action Plan, all levels of government and industry have committed to significantly increasing their use of RWPs
National Waste Policy (2018)	Australian Government	The Australian government has committed to considering environmental sustainability when purchasing goods and services
Environment Protection and Biodiversity Conservation Act 1999 (Australian Government 2020a)	Australian Government	Australian Government agencies to include information about their performance on Ecologically Sustainable Development principles in their Annual Reports
Environmental Purchasing Guide 2003 (Australian Government 2003)	Australian Government	EP can achieve several benefits, such as reducing waste (which can reduce waste disposal costs)

The Australian government established its first sustainable procurement policy in 2003 (Commonwealth of Australia 2003). This policy establishes the key areas for government agencies to consider when evaluating the environmental implications of their procurement activities. However, the guideline provides no guidelines to procure RWPs. The policy was revised in 2020 (Australian Government 2020b) and a five-step approach for integrating sustainability into the procurement processes of Australian organisations were introduced. These steps are as follows: (1) plan the procurement (identify the need, identify the sustainability outcomes, assess the risks and opportunities and engage in market research and engagement); (2) approach the market (specify sustainability requirements, develop key performance indicators and establish sustainability evaluation criteria); (3) evaluate and engage (assess tender responses, debrief unsuccessful tenderers); and (4) report and manage (monitor sustainable compliance, performance) (identify opportunities to improve, monitor and track progress and share your experience). In 2013, the federal government published a report on sustainable procurement that provides an overview of the Australian Government’s procurement policies and operations in 2010–2011 and 2011–2012 that embody sustainable procurement concepts and practices (Australian Government 2013).

The main national waste policy published in 2018, proposes a set of strategies to reduce waste generation through avoidance, reduction, recycling and reuse of waste resources (National Waste Policy 2018). Notably, the policy emphasises the importance of using the concepts of a CE to promote the innovative and recurrent use of existing resources. For this to happen, this policy prioritises two strategies relating to improved sustainable procurement in Australia: Strategy 8 (government sustainable procurement) and Strategy 9 (sustainable procurement by businesses and individuals). These two strategies encourage public and private organisations to focus on the increased procurement of RWPs. In addition, through the 2019 National Waste Policy Action Plan Plan (Australian Government 2019), the federal government defines a number of critical initiatives to achieve the waste minimisation targets, including a recommendation to strengthen governments’ purchasing power for recycling. The Action Plan presupposes that sustainable procurement can increase demand for RWPs relative to virgin materials. Furthermore, it fosters innovation and investment in recycling to fulfil the need of emerging markets, supports local jobs and businesses by preserving the value of RWPs and promotes behaviour change across the economy. In addition to the policies and standards listed above, each government agency has internal policies that promote conscientious procurement concerning resource efficiency and the use of RWPs. In addition to each of the policies and guidelines tabulated above, each government organisation has internal policies that encourage conscious procurement involving themes such as resource efficiency and the use of RWPs.

#### Jurisdictional Guidelines, Policies and Waste Strategy Documents

ACT, NSW, SA, Qld, Vic and WA developed a sustainable procurement guideline among the Australian states and territory governments. These guidelines are linked with several other jurisdictions’ regulations and policies. For instance, the Local Government Act 1993 (NSW), Local Government (General) Regulation 2005 (NSW), NSW Procurement Policy Framework for NSW Government Agencies, Tendering Guidelines for NSW Government 2009 and Local Council’s Policies and Vision Statements all provide legal support for the implementation of the Sustainable Procurement Guide: for local government in NSW (Local Government NSW 2021). Furthermore, procurement of RWPs should comply with material specifications developed and administrated by public agencies in each state/territory. Among the jurisdictional policies, the Victorian Recycled First Policy 2021 is the most relevant driver for using RWPs. Table 7 summarises the jurisdictional sustainable procurement guidelines and their primary objectives.

**Table 7:** Sustainable procurement in state and territory guidelines and policies

State/territory	Guideline policy	Issuing organisation	Policy objective/s
ACT	Sustainable Procurement Policy 2015 (ACT Government 2015)	ACT Government	• Advises that waste should be looked at as a resource opportunity where products can be re-introduced into another product lifecycle
NSW	Sustainable Procurement Guide: for local government in NSW (Local Government NSW 2021)	Local Government NSW	• Provide general advice on how to procure goods and services sustainably
SA	SA Sustainable Procurement Guideline (SA Government 2012)	SA State Government	• Urges the state procurement experts to consider both the end-of-life disposal of assets and also the waste produced by these products and services throughout their life
Vic	Social Procurement Framework Recycled First	Vic Government	• Social Procurement Framework: incorporates sustainable procurement practices and includes requirements of RWPs, waste management and energy consumption for (government agencies) buyers • Recycled First policy: outlines that all bidders on major transport projects will be required to demonstrate how they will optimise recycled or reused Vic’s materials. Provides the government with data on RWPs for a better understanding of the supply chain
Qld	Integrating sustainability into the procurement process (Queensland Government 2018)		• Highlights the opportunities and strategies that exist to address environmental and social impacts during procurement planning; during supplier engagement; and through the management of supply arrangements, including measurement and reporting
WA	Guide to Sustainable Procurement 2017 (WA Department of Finance 2017)	Department of Finance	• Seeks to reduce waste and by-products (e.g. waste avoidance, reuse, use of RWPs or products with recycled content, recycling and resource recovery)

This section analyses the jurisdictional waste management strategy documents (WMD) to provide an overview of each state and territory’s government regarding the implementation of sustainable procurement of RWPs and the development of end markets. Some state and territory WMDs highlight that sustainable procurement can play a crucial part in the waste management of Australian jurisdictions. A word count analysis was used to evaluate the position of sustainable procurement in waste strategy documents in different states and territories. Three keywords used for this analysis were ‘sustainable procurement’, ‘government procurement’ and ‘purchasing power’. The analysis suggests that less than 2% of study waste strategy documents content referenced sustainable procurement. Among the states and territories, WA and SA had the largest frequency of the study keywords; these two documents were recently published, i.e. 2020 and 2021, respectively. Currently, Vic and Tasmania (Tas) do not possess a WMD at present. Tas instead produced a Draft Waste Action Plan in 2019 to reinforce waste management practices throughout the state, but it does not mention the influence of sustainable procurement on waste management. SA, Queensland and WA’s WMDs place the most importance on sustainable procurement in terms of market development. Table 8 is a summary of statements regarding the consideration of sustainable procurement in WMDs that result in the creation of demand for RWPs.

**Table 8:** Sustainable procurement in state and territory waste strategy documents

State/territory	Strategy document	Sustainable procurement	Count (per.) of reference
Australian Capital Territory (ACT)	ACT Waste Management Strategy 2011–2025 (ACT Government 2011)	• Government procurement is recommended as a strategy (2.6) to recover resources fully • The ACT Government can use sustainable procurement principles to provide a market driver for increased use of RWPs in the goods and works that it procures. It can also encourage service suppliers to RWPs where practical • The ACT Government will review the specifications used for government tendering to identify where recyclable alternatives can replace non-recyclable materials, for example, in the tendering of construction and landscaping projects • As part of its proposed Carbon Neutral ACT Government Framework, the ACT Government will pursue additional sustainable procurement practices	12 [0.08%]
Northern Territory (NT)	Waste Management Strategy for the Northern Territory 2015–2022 (NT EPA 2015)	• No mention of sustainable procurement	0 [0%]
NSW	NSW’s Waste and Sustainable Materials Strategy 2041 (Department of Planning 2021)	• Indicates the opportunities to increase government procurement of local RWPs • The government will report annually on the use of RWPs in government procurement and its associated impact on emissions and waste reduction • The government will publish a directory of RWPs suppliers and a register of upcoming government infrastructure and construction projects that will procure RWPs	6 [0.05%]
Qld	Waste Management and Resource Recovery Strategy (Queensland Government 2021)	• Under Strategic priority 3 (building economic opportunity) one government action is to consider how procurement can stimulate demand for RWPs manufactured in Qld • The Qld’s Government will work with local government and the waste management and resource recovery sector to develop a consistent procurement contract framework for waste management and resource recovery services • Local governments should support the Qld’s Government by adopting national or state standards for RWPs in procurement to help stimulate demand for products containing RWPs	4 [0.08%]
SA	SA’s Waste Strategy 2020–2025 (SA Government 2020)	• Investigate barriers to sustainable procurement and identify measures to overcome these • Investigate financial instruments, policies and other measures that would aid in providing a level playing field for local manufacturing of RWPs • Advocate for State Government and local government adoption of sustainable procurement and ‘buy-back’ policies • Identify and recommend priority recovered materials and RWPs to be mandated for use in the government and industry procurement system • Develop successful procurement case studies demonstrating the benefits of using RWPs • Identify relevant training needs for procurement practitioners and develop tools for capacity building in sustainable procurement • Collaborate in and advocate for nationally consistent standards and/or frameworks for the requirement of RWPs in government procurement • Support the development of accredited testing for product standards and performance to increase confidence in the quality of remanufactured products • Develop government fit-out requirements to support increased resource recovery and material reuse and repurpose • Ensure a robust regulatory environment that supports local market development for RWPs • Include RWPs measures in government infrastructure projects • Develop monitoring and reporting mechanisms for sustainable procurement • Develop strategies to develop economically viable products from RWPs	22 [0.1%]
Tas	Draft Waste Action Plan 2019 (Tasmanian Government 2019)	• Boost demand for RWPs through the adoption of sustainable procurement practices across State and local government	3 [0.04%]
Vic	NA	• NA	NA
WA	WA Waste Avoidance and Resource Recovery Strategy 2030 (Waste Authority 2020)	• The document places an increased focus on promoting procurement decisions that prefer local markets and play a role in supporting the development of a remanufacturing industry within WA • Local, State and Commonwealth governments can influence, educate and inform, and be significant consumers whose purchasing decisions and procurement policies can have very positive impacts and influence. They have important legislative and regulatory roles and develop and implement strategies. Australia is also part of global action on waste management • Headline strategy: Implement sustainable government procurement practices that encourage greater use of RWPs and support local market development	17 [0.13%]

### Public State Agencies Involved in Sustainable Procurement of RWP

**Fig. 4:**
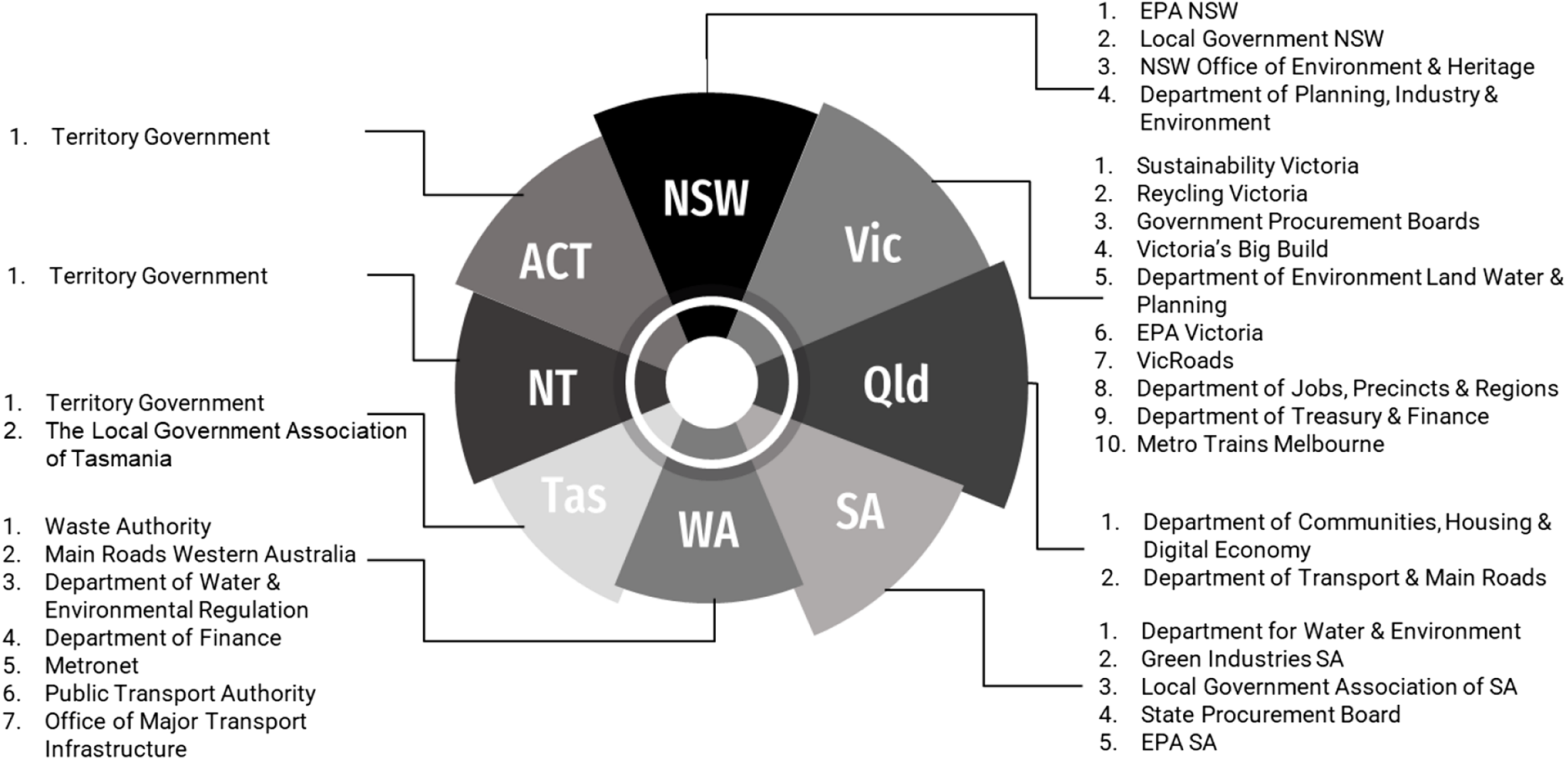
State and territory public agencies are currently involved in the sustainable procurement of waste materials

In each state and territory, various public organisations are directly or indirectly engaged in the procurement of RWPs in construction projects. The following figure shows the analysis of the state agencies that are responsible for the effective use of RWPs in construction projects (Fig. 4). As can be seen, Vic and WA have the largest number of agencies involved in the utilisation of these products.

### Sustainable Procurement Initiatives in Australia

Some states in Australia have begun identifying suppliers of RWPs in order to assist the government organisations in identifying sustainable procurement possibilities. Current initiatives in Vic and WA aiming to increase the use of RWPs through sustainable procurement include the following:

#### Buy Recycled

In 2020, Recycling Victoria as a waste regulatory body was established to strengthen the state’s waste and recycling sector (Victoria State Government 2020). Recycling Victoria in collaboration with Sustainability Victoria launched an online directory (Buy Recycled) that features local RWPs and helps the Vic government and public to procure them. Notably, the initiative supports the state and local government procurers to have a better access to available RWPs across the state. To ensure the successful implementation of RWPs sustainable procurement, this initiative requires the supplier to meet the following five requirements:

- Products must have some amount of recycled content, but otherwise, there is no minimum RWPs requirement.
- Products must be certified against any claims made. For example, a third-party Environmental Product Declaration (EPD).
- Products must meet all legislative and regulatory requirements.
- Products must meet obligations regarding environmental claims under the ACCC Competition and Consumer Act 2010.
- Any claims must meet the ACCC Green marketing and the Australian Consumer Law guidelines.

Furthermore, Recycling Victoria aims to promote the use of RWPs in the state’s transportation infrastructure projects by implementing the Recycled First Policy (ecologiQ 2020). ecologiQ is commissioned to support the objectives of Recycled First Policy and the Buy Recycled initiative to overhaul the state’s recycling sector. Also, ecologiQ has developed RWPs reference guides for road and rail-specific applications and ancillary infrastructure. The guides are an ideal starting point for information about different types of RWPs available in Vic and the current specifications for their use.

#### Advisory System for Processing, Innovation and Resource Exchange (ASPIRE)

ASPIRE is another initiative related to sustainable procurement that was developed by the Commonwealth Scientific and Industrial Research Organisation (CSIRO) under the Vic’s State Government’s Digital Futures Fund in partnership with several Vic’s councils; its operation officially commenced in 2018 (King et al. 2020). ASPIRE intelligently matches businesses with potential purchasers or recyclers of waste by-products. This system requires patrons to enter details about the type and quantity of their exchangeable inputs and waste materials (outputs). ASPIRE’s Supply Chain Options Model uses this data to determine optimal sources and destinations for the materials, including options for aggregation with other local businesses, and appropriate investment opportunities such as compactors for low-density waste materials and local recyclers. ASPIRE is deployed using existing established council and manufacturing business networks and supports local government business sustainability programs. It captures and codifies small-to-medium enterprise (SME) material inputs, outputs (waste and by-products) and processes and has a powerful optimisation model that takes this data and provides an SME user with: (a) suggested business-to-business (B2B) resource matches, both substitute inputs or sources and output destinations, (b) personalised search results to support the suggested matches and (c) case studies for related resource matches.

#### Roads to Reuse (RtR)

In WA, the Waste Avoidance and Resource Recovery Strategy (Waste Authority 2020) governs the evolution of the C&D waste sector which includes defining actions and waste management actions. The actions are operationalised in a platform called Waste Forum which is a multidisciplinary sustainability organisation that was established by Main Roads in 2018 and has produced exceptional results via its commitment to innovation and collaboration. The forum includes the WA Department of Water and Environmental Regulation (DWER), Waste Authority, and Main Roads WA representatives, as well as waste and recycling sector specialists. This forum encourages sustainable purchasing in WA (Feng et al. 2021) through supporting innovative initiatives such as the R2R program. R2R program is the result of a collaboration between the WA Waste Authority, the DWER, and WA local governments, and it intends to enhance the use of RWPs in state-wide public projects by financing pilot projects. Table 9 displays the utilisation of CRC in road construction in WA.

**Table 9:** Statistics on the use of CRC in civil construction projects facilitated by the RtR program

Project	Original target	CRC (T) 2019/20	CRC (T) 2020/21	Forecast CRC
Armadale Road to Northlake Road Bridge	17,355		29,014	0
High St/Stirling Hwy	12,000		12,236	0
Murdoch Drive Connection	4000	7287	0	0
Kwinana Freeway Northbound Widening	16,000	24,263	0	0
Leach—Welshpool	8900	0	2351	6549
Karel Avenue	0	3332	857	0
Tonkin Gap	20,000		3531	20,000
Total		34,882	46,132	

Source: Main Roads WA (cited in Shooshtarian et al. 2022b)

In addition, Les Merchant, Manager of Materials Engineering Pavement and Surfacing for Main Roads WA, reported that approximately 65 kt of crushed glass, 34 kt of CRC, 2.4 kt of crumbed rubber and 20 kt of rubber were utilised in WA infrastructure developments in 2020 as a result of this project (Merchant 2021). Intriguingly, the context enables the latter group to control the entire process to guarantee that impending obstacles, as indicated by them, are effectively addressed. The Waste Forum provides a platform for government and business to communicate in order to develop the capacity to decide on ways to review product specifications for RWPs. The platform unites recycling businesses to market and make the product specifications known to all parties participating in the CRC supply chain. Periodically, in partnership with the network’s industry, the forum arranges training courses to educate stakeholders on the requirements of RWPs. This networking has allowed government organisations to engage with other waste groups, such as the Infrastructure Sustainability Council of Australia (ISC) WA Working group, to gain a more holistic understanding of the issues.

### Evidence of Sustainable Procurement

This section evaluates the impact of sustainable procurement policies and initiatives by examining the quantities of RWPs utilised in Australian construction projects. Multiple pilot studies and actual projects are currently underway in Australia; however, the results of the application of RWPs have not yet been documented. Therefore, this analysis only included completed or almost finished projects. The greatest market for RWPs is now infrastructure projects, where the government’s purchasing power is leveraged to increase the use of RWPs. Table 10 provides a summary of important case studies with reportable environmental benefits in the domain of public infrastructure.

**Table 10:** Quantitative summary of sustainable procurement induced utilisation of RWPs in various infrastructure projects

Project specifications			Sustainability outcome	Ref
Location	Name	Duration		
Melbourne, Vic	Dingley Bypass	2014–2016	269 kilo tonnes (kt) of crushed concrete and recycled sand were supplied throughout the 2-year project. The application of these products resulted in 23 less kt used due to the density savings compared to quarried rock and 770 fewer truck movements. In addition, 1.6 kt of CO2 has been prevented from entering the atmosphere	(Alex Fraser Group 2016)
Melbourne, Vic	The Kororoit Creek road level crossing removal	2017–2018	The project used 1.3 kt of recycled glass sand at the project sites. The cost of the recycled glass sand used in this project was about half the price of virgin material due to shorter transport distances. It is also safer to handle, as it presents a lower respiratory hazard than traditional sand	(Victoria State Government 2019)
Perth, WA	Armadale Road to Northlake Road Bridge	2019–2021	This project that was facilitated by the R2R program used 29 kt of CRC, which equates to almost one-third of the permanent road subbase coming from RWPs Furthermore, 14 kt of fill material was imported from nearby projects, resulting in a 10% increase of reused fill. More than 5% of all asphalt laid incorporated reclaimed asphalt materials, totalling 2.5 kt of RWPs in asphalt pavement. All retaining walls were constructed from Eco-Blocks, which are local RWPs that have been crushed, graded and repurposed	(cited in Shooshtarian et al. 2022a)
Sydney, NSW	Paramatta Light Rail	2019–present	More than 15,000 m of a single rail, 13,650 rail sleepers, 13 km of overhead wires and the track ballast from a closed rail line were used to construct this project. The RWPs will provide approximately 30% of the track required along the 12-km light rail route	(NSW Government 2022)

## Discussion

### Model Development and Recommendations

**Fig. 5:**
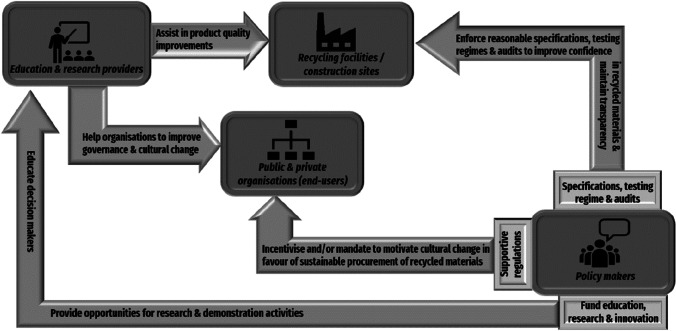
A proposed model to encourage the implementation of sustainable procurement in the BE sector

To facilitate the adoption of sustainable procurement, a model was created that warrant the key stakeholders’ contribution. This model (Fig. 5) is based on the research findings on the primary enablers and barriers of sustainable procurement practice, as well as an assessment of several pertinent policy frameworks. As stated previously, the analysis revealed that the two major barriers are ‘the lack of supportive organisational culture’ and ‘uncertainty about RWPs quality’; whereas the two major enablers are ‘developing clear and supportive regulations’ and ‘maintaining transparency and good governance’.

In the BE sector, the lack of supportive organisational culture can diminish the efforts made to procure RWPs. Due to the novelty of the usage of RWPs in certain applications, the related technical and environmental concerns may diminish the desirability of these materials. Furthermore, fresh convictions are often not easily ingrained in an organisation characterised by decades of stagnation (Cheng et al. 2018). Therefore, it is essential that these companies’ leaders (decision makers) participate in the organisation’s cultural transformation process, as they are responsible for selecting the approach to implement sustainable procurement (Wong et al. 2016). Organisational transformation for a supportive environment is driven by future thinking and circularity that are based on the education of key stakeholders. Many research studies have indicated that the uncertainty around the quality of RWPs is a major barrier to their wide procurement. A part of the issue might be linked with low advances in manufacturing RWPs as indicated by Bougrain (2017) in the case of recycled concrete in France. One study in Australia (Udawatta et al. 2015a) reports that the BE experts believed that some RWPs could not easily be used as new materials and sometimes there is no guarantee of their quality Thus, government funding is necessary to encourage meaningful research and innovation in order to build high-quality RWPs that can be shown in pilot construction projects. Many of the proposed strategies for the effective execution of RWP’s sustainable procurement depend on the existence of clear and supportive regulations. It is often argued that unsupportive regulations, combined with complicated and difficult-to-achieve standards and materials specifications, are further reasons that discourage the procurement of RWPs in construction projects (Knoeri et al. 2011; Park and Tucker 2017). For this reason, the policy makers play a crucial role in ensuring that regulations are in favour of the procurement of recycled materials.

As depicted in Fig. 5, the four factors mentioned above are the building blocks of the proposed model. The proposed model and relate to five stakeholder groups: policymakers, owners of recycling facilities, construction managers, end-users (public and private sectors) and education and research providers. Depending on the circumstances, some or all of the components of this model can be utilised by other nations to boost the circularity of their BE sector.

Drawing on the model above, the following recommendations are provided to improve the application of sustainable procurement policy and practice in the BE sector. These recommendations highlight the important role of policymakers and authorities to encourage different stakeholders to consider sustainable procurement through the following opportunities:

- Government organisations, such as Sustainability Victoria in Vic, to provide funding opportunities to reinforce research and innovation (R&I) activities that would result in increase on the quality of RWPs
- Local governments to foster collaboration with research institutes, such as CSIRO and universities, to carry out demonstration projects exhibiting the performance and reliability of RWPs to end-users
- Education authorities such as the Tertiary Education Quality and Standards Agency (TEQSA) to stimulate educational programs to build knowledge, skills and capabilities necessary to develop resilient supply chains optimised for the increased use of RWPs by end-users
- Regulatory bodies, such as state EPA’s, to modify policy frameworks that encourage or mandate (sustainable) procurement of RWPs in public and private construction projects
- Government-funded industry associations, such as ISC and Green Building Council of Australia (GBCA) to create a reasonable set-up for specifying, testing and auditing RWPs that will result in the production of consistent and acceptable material quality
- State governments to guide waste producers and recycling facilities to provide transparency in their waste material handling and processing

### Research Contribution

This work adds to the development of informed procurement policies, the theory of sustainable procurement and the practice of waste management in the BE sector. According to the findings, the BE sector lacks informed policies with the capacity to encourage players to implement sustainable procurement practices. The model proposed and analytical findings establish the groundwork for policy adjustments with measurable sustainable outcomes. As the first review study conducted in Australia, the study also increases local knowledge and awareness of a crucial CE implementation strategy in the BE sector. This contribution is made by identifying the significant challenges and enablers of sustainable procurement, as well as exploring its benefits and guiding principles. In conclusion, the research reveals the modifications necessary for the sector to optimise its procurement methods in favour of RWPs.

## Conclusions

In the Australian BE sector, the issue of C&D waste has become a cause of concern. Sustainable procurement as a policy strategy, among other techniques, can alter the status quo of the C&D waste management system and aid the Australian BE sector in adopting a CE. The purpose of this review was to assess the current condition of sustainable procurement in the Australian BE sector. The study adopted a mixed-method research methodology based on existing data that partially reflect the current state of RWP procurement factors affecting sustainability. These primarily consist of the Australian policy framework at both the national and state levels, existing sustainable procurement initiatives, the evidence for the use of RWPs through sustainable procurement, as well as main enablers and hurdles.

The findings reveal that the two most significant obstacles are the lack of supportive organisational culture and uncertainty about RWP quality. In addition, implementing clear and supporting regulations and maintaining transparency and good governance are identified as the two most important enablers. Furthermore, the study developed a model that assists stakeholders in finding opportunities for adopting sustainable procurement principles at the policy formulation, education and procurement stages. In order to achieve this objective, a set of recommendations is provided to foster collaboration between five stakeholders (policymakers, research and education providers, end-users, recycling facility owners and project managers in the BE sector) in order to increase the likelihood of RWPs in construction project material procurement. The primary constraint of this review article is the lack of secondary data, which prevents a comprehensive evaluation of the effectiveness of sustainable procurement in the BE industry. To mitigate the impact of this constraint, a comprehensive analysis of sustainable procurement evidence in the BE was done. This enquiry focused on academic literature, government and industry reports and pertinent policies. It is suggested that future studies investigate the attitudes of key stakeholders regarding the use of sustainable procurement in order to strike a balance between competing interests and make this approach standard in the sector’s procurement planning and practise.

## Data Availability

All data generated or analysed during this study are included in this published article.

## Abbreviations

ACT: Australian Capital Territory
APCC: Australasian Procurement and Construction Council
B2B: Business to business
BE: Built environment
C&D waste: Construction and demolition waste
CE: Circular economy
CRC: Crushed recycled concrete
CSIRO: Commonwealth Scientific and Industrial Research Organisation
EPA: Environmental Protection Agency
EPD: Environmental product declaration
EPP: Environmentally preferable purchasing
ESD: Ecologically sustainable development
GBCA: Green Building Council of Australia
GDP: Gross domestic product
GHG: Greenhouse gases
GPP: Green Public Procurement
ILO: International Labour Organization
ISC: Infrastructure Sustainability Council of Australia
kt: Kilo tonne
Mt: Million tonnes
NSW: New South Wales
NT: Northern Territory
OECD: Organisation for Economic Cooperation and Development
Qld: Queensland
RA: Recycled aggregate
RtR: Roads to reuse
RWP: Recycled waste products
SA: South Australia
SME: Small-to-medium enterprise
Tas: Tasmania
UNEP: UN Environment Program
Vic: Victoria
WA: Western Australia
WMD: Waste management document
